# Deimination Protein Profiles in *Alligator mississippiensis* Reveal Plasma and Extracellular Vesicle-Specific Signatures Relating to Immunity, Metabolic Function, and Gene Regulation

**DOI:** 10.3389/fimmu.2020.00651

**Published:** 2020-04-28

**Authors:** Michael F. Criscitiello, Igor Kraev, Lene H. Petersen, Sigrun Lange

**Affiliations:** ^1^Comparative Immunogenetics Laboratory, Department of Veterinary Pathobiology, College of Veterinary Medicine and Biomedical Sciences, Texas A&M University, College Station, TX, United States; ^2^Department of Microbial Pathogenesis and Immunology, College of Medicine, Texas A&M Health Science Center, Texas A&M University, College Station, TX, United States; ^3^Electron Microscopy Suite, Faculty of Science, Technology, Engineering and Mathematics, Open University, Milton Keynes, United Kingdom; ^4^Department of Marine Biology, Texas A&M University at Galvestone, Galveston, TX, United States; ^5^Tissue Architecture and Regeneration Research Group, School of Life Sciences, University of Westminster, London, United Kingdom

**Keywords:** peptidylarginine deiminases, protein deimination/citrullination, American alligator (A*lligator mississippiensis*), extracellular vesicles, immunity, metabolism, antimicrobial/antiviral

## Abstract

Alligators are crocodilians and among few species that endured the Cretaceous–Paleogene extinction event. With long life spans, low metabolic rates, unusual immunological characteristics, including strong antibacterial and antiviral ability, and cancer resistance, crocodilians may hold information for molecular pathways underlying such physiological traits. Peptidylarginine deiminases (PADs) are a group of calcium-activated enzymes that cause posttranslational protein deimination/citrullination in a range of target proteins contributing to protein moonlighting functions in health and disease. PADs are phylogenetically conserved and are also a key regulator of extracellular vesicle (EV) release, a critical part of cellular communication. As little is known about PAD-mediated mechanisms in reptile immunology, this study was aimed at profiling EVs and protein deimination in *Alligator mississippiensis*. Alligator plasma EVs were found to be polydispersed in a 50–400-nm size range. Key immune, metabolic, and gene regulatory proteins were identified to be posttranslationally deiminated in plasma and plasma EVs, with some overlapping hits, while some were unique to either plasma or plasma EVs. In whole plasma, 112 target proteins were identified to be deiminated, while 77 proteins were found as deiminated protein hits in plasma EVs, whereof 31 were specific for EVs only, including proteins specific for gene regulatory functions (e.g., histones). Gene Ontology (GO) and Kyoto Encyclopedia of Genes and Genomes (KEGG) enrichment analysis revealed KEGG pathways specific to deiminated proteins in whole plasma related to adipocytokine signaling, while KEGG pathways of deiminated proteins specific to EVs included ribosome, biosynthesis of amino acids, and glycolysis/gluconeogenesis pathways as well as core histones. This highlights roles for EV-mediated export of deiminated protein cargo with roles in metabolism and gene regulation, also related to cancer. The identification of posttranslational deimination and EV-mediated communication in alligator plasma revealed here contributes to current understanding of protein moonlighting functions and EV-mediated communication in these ancient reptiles, providing novel insight into their unusual immune systems and physiological traits. In addition, our findings may shed light on pathways underlying cancer resistance, antibacterial and antiviral resistance, with translatable value to human pathologies.

## Introduction

Alligators are crocodilians, with two living species, the American alligator (*Alligator mississippiensis*) and the Chinese alligator (*Alligator sinensis*). Alligators are long-lived ancient animals and, alongside crocodiles, are among the few species who endured the Cretaceous–Paleogene extinction event. Crocodilians appeared ~240 million years ago, during the Middle Triassic. Although crocodilians are similar in appearance to other reptiles, they are only distantly related to lizards and belong to the closest extant relatives of birds, therefore occupying an important evolutionary position (1–3). Alligators can endure and occupy unsanitary environments, withstand radiation of high levels, and be routinely exposed to heavy metals but are rarely reported to develop cancer (4). With long life spans and unusual immunological characteristics, including unique antimicrobial responses (5–8), antiviral activity against enveloped viruses, including HIV (9) and low metabolic rate (10), crocodilians may hold information for molecular pathways underlying such unusual physiological traits.

Peptidylarginine deiminases (PADs) are a group of calcium-dependent enzymes that posttranslationally convert arginine into citrulline in target proteins in an irreversible manner (11). Such calcium-mediated deimination/citrullination can lead to structural and sometimes functional changes in target proteins and therefore affect protein function (12, 13). A range of proteins known to undergo this posttranslational modification belong to cytoplasmic, nuclear, and mitochondrial targets, and therefore, depending on which target proteins are modified, deimination can for example contribute to the generation of neo-epitopes as well as affecting gene regulation (14–22). Such posttranslational changes in proteins may also allow for protein moonlighting, an evolutionarily acquired phenomenon facilitating proteins to exhibit several physiologically relevant functions within one polypeptide chain (23, 24).

PADs and associated protein deimination are crucial players in cancer and autoimmune and neurodegenerative diseases (17, 19, 20, 22, 25). PADs have received particular attention due to roles in cancer (20, 22, 26, 27), rheumatoid arthritis (28–33), multiple sclerosis (34–38), as well as due to their contribution to skin physiology and diseases (39). PADs have furthermore been shown to play crucial roles in hypoxia and CNS regeneration (40–44), and roles for PAD2 in promotion of oligodendrocyte differentiation and myelination have been shown (45). PAD-mediated mechanisms have also been related to aging (46, 47). Importantly, PADs have also been implicated in infection, including sepsis and endotoxemia (48–55). Roles for PADs in tissue remodeling and immunity have also recently been described (56–58). PADs have furthermore been identified as important regulators of the release of extracellular vesicles (EVs) (27, 59–62). EVs participate in cellular communication via transfer of cargo proteins and genetic material and can be isolated from most body fluids (20, 63–66). As EV cargo is comprised of a large range of proteins, enzymes, and genetic material, characteristic of the cells of origin, EV signatures can be useful biomarkers and easily isolated from a range of body fluids, including serum and plasma (67, 68). While work on EVs has largely focused on human pathologies, an increasing body of comparative studies with respect to EVs and EV cargo has been performed in a range of taxa, including by our group (69–80).

PADs have been identified in diverse taxa throughout phylogeny, from bacteria to mammals. In mammals, five tissue-specific PAD isozymes with deimination activity are described: three in chicken, one in bony and cartilaginous fish (14, 56, 58, 76, 81), and PAD homologs (arginine deiminases, ADI) in parasites (82), fungi (83), and bacteria (62, 84). While in the American alligator three PADI genes have been reported (PADI1, Gene ID: 102574884, Protein ID: XP_014457295.1; PADI2, Gene ID: 102575591, Protein ID: XP_019355592.1; PADI3, Gene ID: 102574651, Protein ID: XP_014457295.1), no studies have hitherto been carried out on PAD protein function and putative physiological relevance for PAD-mediated posttranslational deimination in crocodilians.

Plasma of the American alligator has previously been evaluated for its exceptional antibacterial activity, including a cathelicidin, which has been identified to show promise against multidrug-resistant *Acinetobacter* without toxicity to eukaryotic cells (8). Blood and plasma biochemistry for baseline physiology assessment has been carried out in alligator (85) as well as corticosterone characterization for assessment of environmental stressors (86), including chronic exposure to selenium (87). To date though, no assessment of EVs has been carried out in crocodilians, and therefore, the roles for EVs in the unusual immune responses and metabolism of alligators remain to be further explored and may provide novel biomarkers.

This current study profiled plasma and plasma-derived EVs for deiminated protein signatures in the American alligator. For the first time, this posttranslational modification is assessed in crocodilians, reporting deimination of key immune, metabolic, and nuclear proteins in alligator and species-specific EV signatures. Our findings provide novel insight into the unusual physiology of crocodilians and may further current understanding of pathways underlying cancer, antiviral and antibacterial resistance.

## Materials and Methods

### Plasma Sampling From Alligator

Blood was collected from the occipital sinus of three healthy young male alligators (weight, 2,538, 2,850, and 2,810 g; snout-vent length, 42.1, 47.1, and 47.2 cm, respectively), and plasma was prepared as previously described (88). In brief, blood samples were collected from the occipital sinus, quickly placed in a non-heparinized microfuge tube, and immediately centrifuged for 2 min at 10,000 g to separate the plasma (88). Sample collection was conducted under Texas A&M Institutional Animal Care and Use Protocol # 2015-0347. Plasma was aliquoted and kept at −80°C until used.

### Isolation of Extracellular Vesicles and Nanoparticle Tracking Analysis (NTA)

Plasma aliquots that had been collected as described above and kept frozen at −80°C were thawed. Plasma EVs were isolated from plasma of individual animals (*n* = 3), using sequential centrifugation and ultracentrifugation in accordance with previously established protocols (61, 76, 79) and according to the recommendations of the minimal information for studies of extracellular vesicles 2018 [MISEV2018; (89)]. For each individual EV preparation, 100 μl of alligator plasma were diluted 1:5 in Dulbecco's phosphate-buffered saline (DPBS, ultrafiltered using a 0.22-μm filter, before use) and then centrifuged at 4,000 g for 30 min at 4°C, to ensure the removal of aggregates and apoptotic bodies. Thereafter, the supernatants were collected and centrifuged further, using ultracentrifugation at 100,000 g for 1 h at 4°C. The EV-enriched pellets were resuspended in 1 ml DPBS and ultracentrifuged again at 100,000 g for 1 h at 4°C. The resulting washed EV pellets were then resuspended in 100 μl DPBS and frozen at −80°C until further use. For EV size distribution profiles and EV quantification, nanoparticle tracking analysis (NTA) was carried out using the NanoSight NS300 system (Malvern, UK), which analyzes particle size based on Brownian motion. The EV samples were diluted 1/100 in DPBS (10 μl of EV preparation diluted in 990 μl of DPBS) and applied to the NanoSight using a syringe pump to ensure continuous flow of the sample. For each sample, five 60-s videos were recorded, keeping the number of particles per frame in between 40 and 60. Replicate histograms were generated from the videos, using the NanoSight software 3.0 (Malvern), representing mean and confidence intervals of the five recordings for each sample.

### Transmission Electron Microscopy

A pool of EVs, isolated from plasma of the three individual animals as described above, was used for morphological analysis using transmission electron microscopy (TEM), according to previously described methods (79, 80). Following isolation, the EVs were frozen at −80°C and used within 3 days for TEM imaging. Before TEM preparation, the EVs were thawed and resuspended in 100 mM sodium cacodylate buffer (pH 7.4), and a drop (~3–5 μl) of the suspension was placed onto a grid with previously glow-discharged carbon support film. After the suspension had partly dried, the EVs were fixed by placing the grid onto a drop of a fixative solution [2.5% glutaraldehyde in 100 mM sodium cacodylate buffer (pH 7.0)] for 1 min at room temperature and washed afterwards by touching the grid to the surface of three drops of distilled water. Excess water was removed by touching the grid to a filter paper. Next, the EVs were stained with 2% aqueous uranyl acetate (Sigma-Aldrich) for 1 min, the excess stain was removed by touching the grid edge to a filter paper, and the grid was let to dry. Imaging of EVs was performed using a JEOL JEM 1400 transmission electron microscope (JEOL, Japan) operated at 80 kV at a magnification of 30,000–60,000 ×. Digital images were recorded using an AMT XR60 CCD camera (Deben, UK).

### Isolation of Deiminated Proteins Using F95 Enrichment

Immunoprecipitation and isolation of deiminated proteins in plasma and plasma-derived EVs was carried out as previously described (76), using the Catch and Release® v2.0 immunoprecipitation kit (Merck, UK) in conjunction with the F95 pan-deimination antibody (MABN328, Merck), which has been developed against a deca-citrullinated peptide and specifically detects proteins modified by deimination/citrullination (90). Alligator plasma pools of the three individual animals (3 ×25 μl) were used for F95 enrichment from whole plasma, while for EVs, total protein was first extracted from a pool of EVs derived from three animals (EV pellets derived from 100 μl plasma per animal), using RIPA+ buffer (Sigma, UK). Following application of RIPA+ buffer, the EVs were incubated on ice for 2 h followed by centrifugation at 16,000 g for 30 min to collect the protein containing supernatant. Thereafter, immunoprecipitation (F95 enrichment) was carried out overnight on a rotating platform at 4°C. F95-enriched proteins were eluted according to the manufacturer's instructions (Merck), using denaturing elution buffer (Merck), and diluted 1:1 in Laemmli sample buffer. The F95-enriched eluates from whole plasma and plasma-EVs were then analyzed by sodium dodecyl sulfate–polyacrylamide gel electrophoresis (SDS-PAGE), followed by Western blotting, silver staining, or liquid chromatography with tandem mass spectrometry (LC-MS/MS).

### Western Blotting Analysis

Alligator plasma and plasma EVs were diluted 1:1 in denaturing 2× Laemmli sample buffer (containing 5% beta-mercaptoethanol, BioRad, UK) and boiled for 5 min at 100°C. Proteins were separated by SDS-PAGE using 4–20% gradient TGX gels (BioRad, UK). Western blotting was carried out using the Trans-Blot® SD semidry transfer cell (BioRad, UK); even transfer was assessed by staining the membranes with PonceauS (Sigma, UK). Blocking was performed for 1 h at room temperature using 5% bovine serum albumin (BSA, Sigma, UK), in Tris-buffered saline (TBS) containing 0.1% Tween 20 (TBS-T; BioRad, UK). Following blocking, the membranes were incubated overnight at 4°C on a shaking platform with the primary antibodies, which were diluted in TBS-T. For the detection of deiminated/citrullinated proteins, the F95 pan-deimination antibody was used (MABN328, Merck, 1/1,000). For the detection of putative PAD proteins in alligator plasma, cross-reaction with antihuman PAD2, PAD3, and PAD4 was assessed using the following antihuman PAD antibodies: anti-PAD2 (ab50257, Abcam, 1/1,000), anti-PAD3 (ab50246, Abcam, 1/1,000), and anti-PAD4 (ab50247, Abcam, 1/1,000), which have previously been shown to cross-react with PAD homologs in a range of taxa (40, 41, 56, 58, 76, 77, 79, 80). Cross-reaction with antibodies against other human PAD isozymes was not assessed in the current study (PAD1 or PAD6). For the detection of deiminated histone H3, the citH3 antibody was used (citH3, Abcam, 1/1,000), which is also a marker of neutrophil extracellular trap formation (NETosis). EV isolates were blotted against two EV-specific markers: CD63 (ab216130, 1/1,000) and Flotillin-1 (Flot-1, ab41927, $\frac{1}{2}$,000), for the characterization of EVs. After primary antibody incubation, the membranes were washed for 3 × 10 min in TBS-T at room temperature (RT) and incubated for 1 h, at RT with horseradish peroxidase (HRP)-conjugated secondary antibodies [antirabbit immunoglobulin G (IgG) (BioRad) or antimouse IgM (BioRad) respectively, diluted 1/3,000 in TBS-T]. The membranes were then washed in TBS-T for 5× 10 min, and positive proteins bands were visualized digitally, using enhanced chemiluminescence (ECL; Amersham, UK) and the UVP BioDoc-ITTM System (Thermo Fisher Scientific, UK).

### Silver Staining

F95-enriched protein eluates from alligator plasma and plasma EVs were silver stained following SDS-PAGE (4–20% gradient TGX gels, BioRad, UK) under reducing conditions. The BioRad Silver Stain Plus Kit (1610449, BioRad, UK) was used, according to the manufacturer's instructions (BioRad) and previously described methods (91).

### Liquid Chromatography With Tandem Mass Spectrometry Analysis of Deiminated Protein Candidates

F95-enriched eluates from alligator plasma and plasma EVs were analyzed by LC-MS/MS as previously described (79, 80). For LC-MS/MS analysis, the F95-enriched eluates were run 0.5 cm into a 12% TGX gel (BioRad, UK), the band cut out, trypsin digested, and subjected to proteomic analysis using a Dionex Ultimate 3000 RSLC nano-UPLC (Thermo Fisher Scientific Inc., Waltham, MA, USA) system and a QExactive Orbitrap mass spectrometer (Thermo Fisher Scientific Inc., Waltham, MA, USA). Peptides were separated by reverse-phase chromatography (flowrate, 300 nl/min) and using a Thermo Scientific reverse-phase nano Easy-Spray column (Thermo Scientific PepMap C18, 2 μm particle size, 100 A pore size, 75 μm i.d. × 50 cm length). Peptides were next loaded onto a precolumn (Thermo Scientific PepMap 100 C18, 5 μm particle size, 100 A pore size, 300 μm i.d. ×5 mm length) for 3 min, from the Ultimate 3000 autosampler, in the presence of 0.1% formic acid, at a 10 μl/min flowrate. Thereafter, elution of peptides from the precolumn onto the analytical column was facilitated by switching the column valve (solvent A = water + 0.1% formic acid; solvent B = 80% acetonitrile, 20% water + 0.1% formic acid). A linear gradient of 2–40% B was employed for 30 min. The LC eluent was sprayed into the mass spectrometer (using the Easy-Spray source, Thermo Fisher Scientific Inc.). Measuring of all *m*/*z* values for eluting ions was performed using an Orbitrap mass analyzer; setting was at a resolution of 70,000 and scanning between *m*/*z* 380–1,500. For automatic isolation and generation of fragment ions by higher energy collisional dissociation (HCD, NCE, 25%), data-dependent scans (Top 20) were employed, in the HCD collision cell. The measurement of resulting fragment ions was then performed using the Orbitrap analyzer, which was set at a resolution of 17,500. Ions with unassigned charge states and singly charged ions were excluded from being selected for MS/MS. Furthermore, a dynamic exclusion window of 20 s was employed. The data were processed postrun, using Protein Discoverer (version 2.1., Thermo Scientific); all MS/MS data were converted to mgf files. For the identification of deiminated protein hits, the files were next submitted to Mascot (Matrix Science, London, UK) and searched against the UniProt *Alligator mississippiensis*_20191104 database (31974 sequences; 16476323 residues) and a common contaminant sequences (123 sequences; 40594 residues). The fragment mass and peptide tolerances were, respectively, set to 20 ppm and 0.1 Da. The significance threshold was set at *p* < 0.05, and the peptide cutoff score was set at 20 (analysis carried out by Cambridge Proteomics, Cambridge, UK).

### Protein–Protein Interaction Network Analysis

For the identification and prediction of putative interaction networks for deiminated proteins identified in alligator plasma and plasma EVs, the Search Tool for the Retrieval of Interacting Genes/Proteins analysis (STRING; https://string-db.org/) was used as previously described (80). Protein networks were built based on the protein IDs and using the function of “search multiple proteins” in STRING, choosing “*Alligator mississippiensis*” as the species database. Settings for the analysis were set at “basic,” and confidence was applied at “medium.” Color lines connecting the nodes indicate the following evidence-based interactions for network edges: known interactions (based on curated databases, experimentally determined), coexpression or protein homology, predicted interactions (based on gene neighborhood, gene fusion, gene co-occurrence), or via text mining.

### Phylogenetic Comparison of American Alligator PADs With Human PADs

Previously reported predicted alligator (*A. mississippiensis*) protein sequences for PAD1 (XP_006259278.3), PAD2 (XP_019355592.1), and PAD3 (XP_014457295.1) isozymes were aligned with human PAD isozyme sequences PAD1 (NP_037490.2), PAD2 (NP_031391.2), PAD3 (NP_057317.2), PAD4 (NP_036519.2), and PAD6 (NP_997304.3), using Clustal Omega (https://www.ebi.ac.uk/Tools/msa/clustalo/). A neighbor-joining phylogeny tree was constructed.

### Statistical Analysis

The histograms and the graphs were prepared using the Nanosight 3.0 software (Malvern, UK) and GraphPad Prism version 7 (GraphPad Software, San Diego, USA). NTA curves represent mean and standard error of mean (SEM), indicated by confidence intervals. STRING analysis (https://string-db.org/) was used for the prediction of protein–protein interaction networks. Significance was set at *p* ≤ 0.05.

## Results

### Characterization of Alligator Plasma EVs

**Figure 1:**
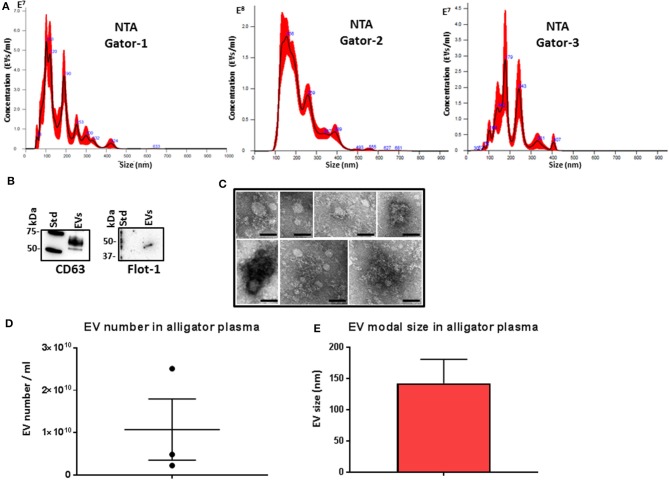
Extracellular vesicle profiling in alligator plasma. **(A)** Nanoparticle tracking analysis shows a size distribution of plasma extracellular vesicles (EVs) from *Alligator mississippiensis* in the size range of mainly 50–400 nm, albeit with some individual variation in EV profiles within these size ranges and peaks at smaller (30 nm) and larger (500 nm) sizes, with main peaks at ~50, 100, 200, 300, and 400 nm. **(B)** Western blotting analysis confirms that alligator EVs are positive for the phylogenetically conserved EV-specific markers CD63 and Flot-1. **(C)** Transmission electron microscopy (TEM) analysis of alligator plasma-derived EVs shows typical EV morphology; scale bar is 100 nm in all figures. **(D)** EV yield in alligator plasma (*n* = 3). **(E)** EV modal size in alligator plasma (*n* = 3).

Plasma EVs were assessed by nanoparticle tracking analysis (NTA) for particle numbers and size distribution using the NanoSight NS300 system, revealing a poly-dispersed population of EVs in the size range of mainly 50–400 nm, albeit with some individual variation in EV profiles within these size ranges and peaks at smaller (30 nm) and larger (500 nm) sizes (Figure 1A). Further characterization of the EVs was performed by Western blotting using the EV-specific markers CD63 and Flot-1 (Figure 1B), and by TEM, confirming typical EV morphology (Figure 1C). Some variation was observed between the three individuals with respect to EV yield (Figure 1D) and modal EV size, which fell in the range of 110–170 nm (Figure 1E).

### PAD Protein Homologs and Deiminated Proteins in Alligator Plasma and Plasma EVs

**Figure 2:**
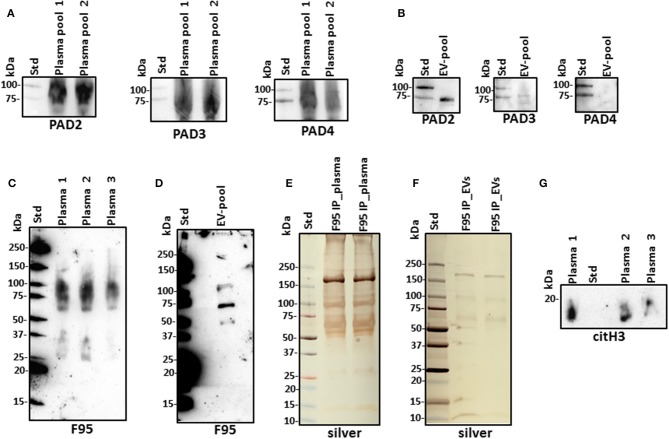
Peptidylarginine deiminases (PADs) and deiminated proteins in alligator plasma and plasma extracellular vesicles (EVs). **(A)** PAD positive bands were identified at the expected size range of approximately 70–75 kDa using the antihuman PAD2-, PAD3-, and PAD4-specific antibodies in alligator plasma. **(B)** Plasma EVs show positive for PAD2 and for PAD3 at lower levels but negative for PAD4 (at expected 70–75 kDa size range), using antihuman PAD isozyme-specific antibodies against PAD2, PAD3, and PAD4, respectively. **(C)** Total deiminated proteins were identified in alligator plasma using the pan-deimination-specific F95 antibody. **(D)** Total deiminated proteins were identified in alligator plasma EVs using the pan-deimination-specific F95 antibody. **(E,F)** F95-enriched IP fraction from alligator plasma **(E)** and plasma EVs **(F)**, shown by silver staining. **(G)** Deiminated histone H3 (citH3) is detected in alligator plasma. The F95-enriched IP fraction derived from a pool of three individual alligator plasma and a pool of plasma EVs (*n* = 3) is shown, respectively.

For assessment of alligator PAD protein homologs, antihuman PAD-isozyme-specific antibodies were used for Western blotting, identifying positive protein bands at an expected ~70–75 kDa size for cross-reaction with antihuman PAD2, PAD3, and PAD4 antibodies in plasma, although this was most prominent for anti-PAD2 (Figure 2A). In plasma EVs, cross-reaction with antihuman PAD2 antibody was prominent, and cross-reaction with antihuman PAD3 was detected at low levels, while the EVs did not show positive against the anti-human PAD4 antibody (Figure 2B). Cross-reaction with other antihuman PAD antibodies (against PAD1 or PAD6) was not tested in the current study. For assessment of total deiminated proteins present in plasma and plasma EVs, the pan-deimination F95 antibody revealed positive bands between 25 and 250 kDa in plasma (Figure 2C) and in EVs mainly in the size range of 50–150 kDa (Figure 2D). The F95-enriched fractions obtained by immunoprecipitation from alligator plasma and plasma EVs were assessed by SDS-PAGE and silver staining, showing protein bands in the size range of 15–250 kDa in plasma and 10–250 kDa in EVs (Figures 2E,F). The presence of deiminated histone H3, also a putative marker of NETosis, was confirmed in alligator plasma in the expected 17–20 kDa size range (Figure 2G).

### LC-MS/MS Analysis of Deiminated Proteins in Alligator Plasma and Plasma EVs

**Figure 3:**
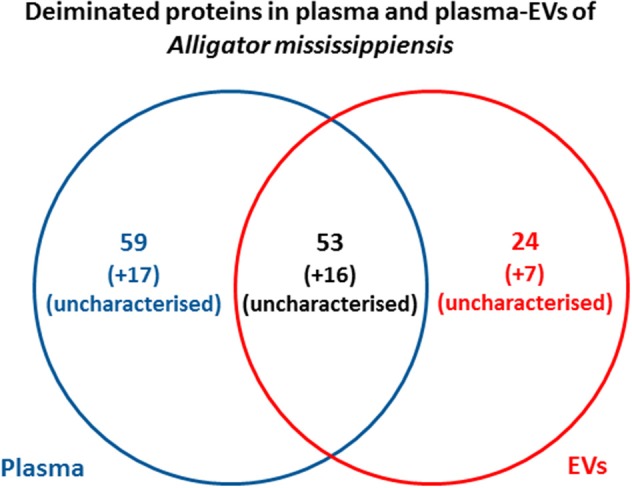
Deiminated proteins identified in alligator plasma and plasma extracellular vesicles (EVs). Species-specific hits identified for deiminated proteins in American alligator plasma and EVs showed overall 145 proteins identified in plasma and 100 in EVs. Of these, 69 protein hits were overlapping, while 76 proteins were specific for whole plasma and 31 for plasma EVs only, respectively.

Protein identification of deiminated proteins in alligator plasma and plasma EVs was carried out using F95 enrichment and LC-MS/MS analysis, searching for species-specific protein hits using the *Alligator mississippiensis* protein database. In plasma, 112 species-specific deiminated protein hits were identified (and further 33 species-specific uncharacterized protein hits) (Table 1 and Supplementary Table 1). In plasma EVs, 77 species-specific deiminated protein hits were identified (and further 23 species-specific uncharacterized protein hits) (Table 2 and Supplementary Table 2). Of the hits identified, 59 proteins were specific for whole plasma only (and an additional 17 uncharacterized alligator protein hits) and 24 proteins for EVs only (with an additional 7 uncharacterized alligator protein hits), while 53 hits overlapped (with an additional 16 unidentified alligator hits) (Figure 3).

**Table 1:** Deiminated proteins in plasma of alligator (*Alligator mississippiensis*), as identified by F95 enrichment and liquid chromatography with tandem mass spectrometry (LC-MS/MS) analysis.

**Protein name**	**Symbol**	**Sequences**	**Total score** **(*p* < 0.05)^†^**
Uncharacterized protein (complement C3)	A0A151NL74_ALLMI	84	5,545
Uncharacterized protein (venom factor)	A0A151NM44_ALLMI	64	4,001
Uncharacterized protein	A0A151NFJ9_ALLMI	55	3,599
Uncharacterized protein	A0A151MJS8_ALLMI	55	3,527
Uncharacterized protein	A0A151NDR9_ALLMI	51	3,205
Serum albumin	A0A151N5S7_ALLMI	45	2,909
Fibrinogen beta chain	A0A151N5S7_ALLMI	37	2,603
Uncharacterized protein	A0A151N583_ALLMI	38	2,432
Fibrinogen gamma chain	A0A151PB79_ALLMI	33	2,280
Alpha-2-macroglobulin-like	A0A151NFQ0_ALLMI	28	2,233
Plasminogen	A0A151M2C0_ALLMI	36	2,185
Plasminogen	A0A151M2C2_ALLMI	36	2,184
Uncharacterized protein	A0A151MZ64_ALLMI	31	2,150
Complement receptor type 1-like	A0A151MS72_ALLMI	27	1,798
Uncharacterized protein	A0A151NDR1_ALLMI	25	1,797
Melanotransferrin	A0A151MJZ7_ALLMI	27	1,766
Kininogen-1	A0A151M678_ALLMI	24	1,693
Hemopexin	A0A151NGB2_ALLMI	23	1,662
Plasma kallikrein isoform B	A0A151PAG7_ALLMI	26	1,627
Complement C5	A0A151NUM2_ALLMI	22	1,426
Uncharacterized protein	A0A151NLW7_ALLMI	20	1,328
Uncharacterized protein	A0A151MYR7_ALLMI	19	1,304
Uncharacterized protein	A0A151MYS3_ALLMI	16	1,191
Uncharacterized protein	A0A151MJK7_ALLMI	19	1,183
Uncharacterized protein	A0A151NAV0_ALLMI	19	1,181
Fetuin-B isoform B	A0A151M7P5_ALLMI	20	1,164
Complement factor H	A0A151NM72_ALLMI	17	1,054
IgGFc-binding protein-like	A0A151MJC3_ALLMI	14	1,043
Vitamin D-binding protein	A0A151N541_ALLMI	19	998
Carbonic anhydrase 6	A0A151N4K1_ALLMI	15	920
Uncharacterized protein	A0A151MYV2_ALLMI	12	911
Uncharacterized protein	A0A151P5P7_ALLMI	15	873
CD5 antigen-like	A0A151PHB7_ALLMI	13	867
Antithrombin-III	A0A151MIW1_ALLMI	13	792
Uncharacterized protein	A0A151MYX2_ALLMI	10	752
Uncharacterized protein	A0A151PB91_ALLMI	9	726
Ovoinhibitor	A0A151MEG6_ALLMI	11	625
Coagulation factor XII	A0A151NM16_ALLMI	8	610
Alpha-1-inhibitor 3-like	A0A151M7F0_ALLMI	11	591
T-cell surface glycoprotein CD8 beta chain	A0A151P975_ALLMI	6	567
Hemoglobin subunit alpha-A	A0A151P678_ALLMI	9	538
Uncharacterized protein	A0A151MYQ9_ALLMI	8	514
Ig lambda chain V-1 region	A0A151P8L7_ALLMI	6	500
Uncharacterized protein	A0A151MZ31_ALLMI	7	484
Heparin cofactor 2 isoform B	A0A151MLS1_ALLMI	9	483
Glutathione peroxidase	A0A151MUD9_ALLMI	8	480
Uncharacterized protein	A0A151MYZ1_ALLMI	6	479
Inter-alpha-trypsin inhibitor heavy chain H2 isoform A	A0A151NPL6_ALLMI	8	459
Ficolin-3	A0A151NG70_ALLMI	8	455
Hemoglobin subunit beta	A0A151PG12_ALLMI	7	450
Ig epsilon chain C region	A0A151MYW1_ALLMI	7	439
Apolipoprotein E	A0A151N3F1_ALLMI	6	430
Complement factor I	A0A151MIQ2_ALLMI	7	424
Fibrinogen C-terminal domain-containing protein	A0A151MF51_ALLMI	5	420
TED_complement domain-containing protein	A0A151N5Z3_ALLMI	6	417
Complement C1q subcomponent subunit B	A0A151MZU2_ALLMI	6	374
Alpha-1-antitrypsin	A0A151P8U9_ALLMI	6	367
Uncharacterized protein	A0A151M7P2_ALLMI	4	351
Coagulation factor XIII B chain	A0A151NCL4_ALLMI	5	333
Alpha-1-antitrypsin	A0A151P8P3_ALLMI	6	322
Alpha-2-antiplasmin	A0A151LY18_ALLMI	6	321
Fructose-bisphosphate aldolase	A0A151MLN4_ALLMI	4	303
Basement membrane-specific heparan sulfate proteoglycan core protein	A0A151MTT0_ALLMI	5	299
Coagulation factor XIII A chain isoform A	A0A151NCL4_ALLMI	7	295
Complement C1q subcomponent subunit C	A0A151N005_ALLMI	4	290
Uncharacterized protein	A0A151MFZ6_ALLMI	5	271
Zinc finger and BTB domain-containing protein 4	A0A151MYL8_ALLMI	5	265
Complement C1q subcomponent subunit A	A0A151MZN0_ALLMI	4	245
Keratin, type I cytoskeletal 19	A0A151PC64_ALLMI	4	234
Haptoglobin	A0A151MVG5_ALLMI	4	228
Ig-like domain-containing protein	A0A151NG86_ALLMI	3	206
C4b-binding protein alpha chain-like	A0A151MAQ5_ALLMI	5	206
Serpin peptidase inhibitor, clade A (Alpha-1 antiproteinase, antitrypsin), member 4	A0A151P987_ALLMI	5	201
Ovostatin-like protein 1-like	A0A151MRY5_ALLMI	3	198
Adiponectin	A0A151M626_ALLMI	2	181
Ig-like domain-containing protein	A0A151M7S2_ALLMI	2	176
Plasma protease C1 inhibitor	A0A151NVG0_ALLMI	3	173
Uncharacterized protein	A0A151NG84_ALLMI	3	173
Stanniocalcin-2	A0A151M6X4_ALLMI	3	168
Pantetheinase	A0A151M3D7_ALLMI	3	162
Uncharacterized protein	A0A151MFZ3_ALLMI	3	155
Retinoic acid receptor responder protein 2	A0A151LYA0_ALLMI	2	161
Protein AMBP	A0A151MF04_ALLMI	2	139
Ig-like domain-containing protein	A0A151P541_ALLMI	2	138
V-set domain-containing T-cell activation inhibitor 1-like	A0A151MVY5_ALLMI	3	137
Protein Z-dependent protease inhibitor	A0A151P8Q1_ALLMI	3	135
Fibrinogen C-terminal domain-containing protein	A0A151MF29_ALLMI	2	134
Ig-like domain-containing protein	A0A151P538_ALLMI	2	133
Vitelline membrane outer layer 1-like protein	A0A151PFR3_ALLMI	3	132
Apolipoprotein A-IV	A0A151LZQ0_ALLMI	2	131
Ig-like domain-containing protein	A0A151NR11_ALLMI	2	131
Keratin, type I cytoskeletal 14	A0A151PC58_ALLMI	3	130
Vitronectin	A0A151NVP9_ALLMI	2	130
Sushi domain-containing protein	A0A151P1M1_ALLMI	2	129
Uncharacterized protein	A0A151MP64_ALLMI	3	126
Disabled-like protein 1 isoform B	A0A151M4I5_ALLMI	2	121
Beta-2-glycoprotein 1	A0A151N2D1_ALLMI	2	120
Ig-like domain-containing protein	A0A151M7W3_ALLMI	1	118
Complement component C8 alpha chain	A0A151M4H1_ALLMI	2	113
Histidine-rich glycoprotein	A0A151M7M7_ALLMI	2	112
Chondroadherin isoform A	A0A151N2Q4_ALLMI	2	109
Ig-like domain-containing protein	A0A151M7N2_ALLMI	1	108
Complement factor H-related protein 3-like	A0A151NBK6_ALLMI	3	108
Uncharacterized protein (apolipoprotein E-like)	A0A151N395_ALLMI	2	105
Ig-like domain-containing protein	A0A151P518_ALLMI	2	104
Alpha-1-antitrypsin-like	A0A151P8V8_ALLMI	2	94
Ig heavy chain V region 6.96	A0A151P441_ALLMI	2	93
Leucine-rich alpha-2-glycoprotein	A0A151MCJ2_ALLMI	2	90
Uncharacterized protein	A0A151MAL5_ALLMI	2	88
Ig-like domain-containing protein	A0A151P518_ALLMI	2	86
Alpha-1-antiproteinase-like	A0A151P8W0_ALLMI	2	82
Insulin-like growth factor-binding protein complex acid labile subunit	A0A151N6V5_ALLMI	2	78
Apolipoprotein B-100	A0A151PIT1_ALLMI	3	77
Properdin	A0A151MYJ4_ALLMI	2	77
Complement component C7	A0A151MX90_ALLMI	2	75
Anionic trypsin-2-like	A0A151NL42_ALLMI	1	75
Angiogenin	A0A151MM94_ALLMI	1	73
*N*-Acetylmuramoyl-l-alanine amidase	A0A151PH98_ALLMI	2	72
Ig-like domain-containing protein	A0A151N4A7_ALLMI	2	72
GRIP and coiled-coil domain-containing protein 2	A0A151NXH0_ALLMI	2	69
Fibulin-1	A0A151P794_ALLMI	2	66
Complement C2	A0A151PIR2_ALLMI	2	63
Selenoprotein P	A0A151MWX0_ALLMI	2	54
Ig-like domain-containing protein	A0A151P973_ALLMI	1	54
Ig-like domain-containing protein	A0A151MRI0_ALLMI	1	53
Pericentrin isoform D	A0A151N9S1_ALLMI	2	53
Uncharacterized protein	A0A151NLI3_ALLMI	2	53
AT-rich interactive domain-containing protein 3B	A0A151MAK9_ALLMI	2	52
Laminin subunit beta-3 isoform A	A0A151MJP2_ALLMI	2	51
Uncharacterized protein	A0A151NFR7_ALLMI	1	51
Signal transducer and activator of transcription	A0A151PC08_ALLMI	2	51
ZZ-type zinc finger-containing protein 3 isoform B	A0A151NJ96_ALLMI	2	50
Ig-like domain-containing protein	A0A151NR16_ALLMI	1	49
WD repeat-containing protein 11 isoform C	A0A151NU67_ALLMI	2	48
Avidin-like	A0A151MRC9_ALLMI	1	47
Complement factor B	A0A151PIT0_ALLMI	1	47
Uncharacterized protein	A0A151MF67_ALLMI	1	46
Complement factor H-like	A0A151NLD7_ALLMI	1	46
Uncharacterized protein	A0A151MT59_ALLMI	1	44
Uncharacterized protein	A0A151NT34_ALLMI	1	44
Sulfhydryl oxidase	A0A151MIB1_ALLMI	1	42
Exostosin-like 1 isoform B	A0A151MMH5_ALLMI	1	42
Putative E3 ubiquitin-protein ligase UBR7	A0A151P8Y0_ALLMI	2	41
Sorbitol dehydrogenase	A0A151MAA7_ALLMI	1	41
Carboxypeptidase B2	A0A151MHY8_ALLMI	1	40
T-complex protein 1 subunit eta	A0A151MYL4_ALLMI	1	40
BTB/POZ domain-containing protein 7	A0A151P8X9_ALLMI	1	39
Citron Rho-interacting kinase isoform B	A0A151N4K3_ALLMI	1	39
Uncharacterized protein	A0A151ND16_ALLMI	1	39
Prickle-like protein 1 isoform A	A0A151PEZ2_ALLMI	1	39
Ras-related protein Rab-17 isoform B	A0A151N9P9_ALLMI	1	38
Uncharacterized protein	A0A151LZX4_ALLMI	1	38
T-lymphoma invasion and metastasis-inducing protein 1	A0A151ME23_ALLMI	1	38
Uncharacterized protein	A0A151NRI7_ALLMI	1	38
Neuronal PAS domain-containing protein 3 isoform A	A0A151NKE6_ALLMI	1	37
Ankyrin repeat domain-containing protein 26 isoform A	A0A151PEV8_ALLMI	1	37
Cadherin-1	A0A151P0T5_ALLMI	1	36
Ig-like domain-containing protein	A0A151NR67_ALLMI	1	34
Uncharacterized protein	A0A151M2C1_ALLMI	1	34
Zinc finger castor-like protein 1 isoform C	A0A151N3T3_ALLMI	1	34
Ubiquitin carboxyl-terminal hydrolase 8 isoform C	A0A151M9E3_ALLMI	1	33
Uncharacterized protein	A0A151P059_ALLMI	1	33
Arf-GAP with coiled coil, ANK repeat, and PH domain-containing protein 1	A0A151N149_ALLMI	1	33
Membrane-bound transcription factor site-1 protease	A0A151MLJ3_ALLMI	1	33
TRAF family member-associated NF-kappa-B activator	A0A151M0A1_ALLMI	1	32
Pyruvate carboxylase, mitochondrial	A0A151PCD5_ALLMI	1	32
Leukocyte receptor cluster member 8	A0A151N898_ALLMI	1	32

*Deiminated proteins in alligator plasma were isolated by immunoprecipitation using the pan-deimination F95 antibody. The resulting F95-enriched eluate was then analysed by LC-MS/MS and peak list files submitted to mascot. Alligator mississippiensis species-specific peptide sequence hits are listed (ALLMI), showing number of sequences for protein hits and total score. Blue highlighted rows indicate protein hits identified in whole plasma only (for full details on protein hits see Supplementary Table 1)*.

† *Ions score is −10 × Log(P), where P is the probability that the observed match is a random event. Individual ions scores >32 indicated identity or extensive homology (p < 0.05). Protein scores were derived from ions scores as a non-probabilistic basis for ranking protein hits*.

**Table 2:** Deiminated proteins in plasma extracellular vesicles (EVs) of alligator (*Alligator mississippiensis*), as identified by F95 enrichment.

**Protein name**	**Symbol**	**Sequences**	**Total score** **(*p* < 0.05)^†^**
Uncharacterized protein (complement C3)	A0A151NL74_ALLMI	62	3,704
Uncharacterized protein	A0A151NFJ9_ALLMI	43	2,719
Serum albumin	A0A151N5S7_ALLMI	32	1,711
Fibrinogen alpha chain	A0A151PBF3_ALLMI	25	1,672
Fibrinogen beta chain	A0A151PC06_ALLMI	25	1,526
Alpha-2-macroglobulin-like	A0A151NFQ0_ALLMI	23	1,486
Uncharacterized protein (venom factor)	A0A151NM44_ALLMI	25	1,411
Fibrinogen gamma chain	A0A151PB79_ALLMI	22	1,305
Uncharacterized protein	A0A151N583_ALLMI	22	1,262
Uncharacterized protein	A0A151MZ64_ALLMI	20	1,221
Kininogen-1	A0A151M678_ALLMI	19	1,213
Hemopexin	A0A151NGB2_ALLMI	18	1,107
Melanotransferrin	A0A151MJZ7_ALLMI	15	906
Uncharacterized protein	A0A151MYS3_ALLMI	13	856
Complement receptor type 1-like	A0A151MS72_ALLMI	15	838
Uncharacterized protein	A0A151MJS8_ALLMI	13	791
Carbonic anhydrase 6	A0A151N4K1_ALLMI	13	736
Uncharacterized protein	A0A151NDR9_ALLMI	13	679
Uncharacterized protein	A0A151MYV2_ALLMI	8	656
Uncharacterized protein	A0A151PB91_ALLMI	8	637
Fetuin-B isoform B	A0A151M7P5_ALLMI	10	576
Carbonic anhydrase 6	A0A151N4K1_ALLMI	10	540
Plasma kallikrein isoform B	A0A151PAG7_ALLMI	11	533
Uncharacterized protein	A0A151P5P7_ALLMI	10	475
CD5 antigen-like	A0A151PHB7_ALLMI	6	469
Keratin, type I cytoskeletal 19	A0A151PC64_ALLMI	7	445
Keratin, type I cytoskeletal 20	A0A151PC95_ALLMI	8	425
Uncharacterized protein	A0A151MAL5_ALLMI	8	407
IF rod domain-containing protein	A0A151LYF4_ALLMI	8	391
Ig lambda chain V-1 region	A0A151P8L7_ALLMI	4	347
Complement factor H	A0A151NM72_ALLMI	5	336
Hemoglobin subunit beta	A0A151PG12_ALLMI	5	326
Keratin, type I cytoskeletal 14	A0A151PC58_ALLMI	5	325
T-cell surface glycoprotein CD8 beta chain	A0A151P975_ALLMI	3	292
Fibrinogen C-terminal domain-containing protein	A0A151MF51_ALLMI	4	285
Complement C5	A0A151NUM2_ALLMI	5	281
Plasminogen	A0A151M2C0_ALLMI	4	259
Complement C1q subcomponent subunit B	A0A151MZU2_ALLMI	4	253
Complement C1q subcomponent subunit C	A0A151N005_ALLMI	3	251
Uncharacterized protein	A0A151MYZ1_ALLMI	5	248
Uncharacterized protein	A0A151NDR1_ALLMI	5	236
Glutathione peroxidase	A0A151MUD9_ALLMI	4	234
Uncharacterized protein	A0A151MJK7_ALLMI	4	227
IF rod domain-containing protein	A0A151LYE5_ALLMI	4	223
Uncharacterized protein	A0A151MYX2_ALLMI	3	208
Complement C1q subcomponent subunit A	A0A151MZN0_ALLMI	3	192
Hemoglobin subunit alpha-A	A0A151P678_ALLMI	4	181
Alpha-1-inhibitor 3-like	A0A151M7F0_ALLMI	4	180
Uncharacterized protein	A0A151NSW8_ALLMI	3	154
Ig-like domain-containing protein	A0A151P538_ALLMI	3	145
Histone H4	A0A151NSZ8_ALLMI	3	130
TED_complement domain-containing protein	A0A151N5Z3_ALLMI	2	126
Ig-like domain-containing protein	A0A151P518_ALLMI	2	126
Uncharacterized protein	A0A151MP64_ALLMI	2	120
Antithrombin-III	A0A151MIW1_ALLMI	2	113
Vitronectin	A0A151NVP9_ALLMI	2	108
HATPase_c domain-containing protein	A0A151NIG2_ALLMI	1	107
Desmin	A0A151MQA4_ALLMI	2	104
Ig-like domain-containing protein	A0A151M7S2_ALLMI	2	103
Heterogeneous nuclear ribonucleoproteins A2/B1	A0A151MQK4_ALLMI	2	100
Ig epsilon chain C region	A0A151MYW1_ALLMI	2	97
Alpha-1-antitrypsin	A0A151P8P3_ALLMI	2	95
Uncharacterized protein	A0A151N405_ALLMI	3	92
l-lactate dehydrogenase	A0A151MCB3_ALLMI	1	87
Uncharacterized protein	A0A151NG84_ALLMI	2	87
Ovostatin-like protein 1-like	A0A151MRY5_ALLMI	2	83
Histone H2A	A0A151LYY4_ALLMI	2	82
Heterogeneous nuclear ribonucleoprotein U	A0A151MHH8_ALLMI	1	80
Tubulin beta chain	A0A151MZH9_ALLMI	2	78
Heparin cofactor 2 isoform B	A0A151MLS1_ALLMI	2	77
Olfactory receptor	A0A151M3L3_ALLMI	1	73
Anionic trypsin-2-like	A0A151NL42_ALLMI	1	72
Glyceraldehyde-3-phosphate dehydrogenase	A0A151M7G4_ALLMI	2	67
Ovoinhibitor	A0A151MEG6_ALLMI	2	67
Glyceraldehyde-3-phosphate dehydrogenase	A0A151MCM1_ALLMI	2	66
Plasma protease C1 inhibitor	A0A151NVG0_ALLMI	2	63
40S ribosomal protein SA	A0A151P2K0_ALLMI	1	61
Triosephosphate isomerase	A0A151MCS3_ALLMI	2	60
Heat-shock protein, mitochondrial	A0A151NA12_ALLMI	1	59
Uncharacterized protein	A0A151PC76_ALLMI	2	59
Actin filament-associated protein 1-like 2 isoform D	A0A151NUD0_ALLMI	2	59
Tr-type G domain-containing protein	A0A151MMV6_ALLMI	1	59
Desmoplakin	A0A151ND53_ALLMI	2	58
Uncharacterized protein	A0A151NLW7_ALLMI	2	57
Ig-like domain-containing protein	A0A151MRI0_ALLMI	1	55
Tubulin alpha chain	A0A151N6Z6_ALLMI	1	53
Fibrinogen C-terminal domain-containing protein	A0A151MF29_ALLMI	1	53
Serpin peptidase inhibitor, clade A (Alpha-1 antiproteinase, antitrypsin), member 4	A0A151P987_ALLMI	1	51
Vitamin D-binding protein	A0A151N541_ALLMI	2	50
60S ribosomal protein L23a	A0A151MUP1_ALLMI	1	47
Tropomyosin alpha-1 chain isoform	A0A151MA07_ALLMI	1	46
Kinesin motor domain-containing protein	A0A151M9A9_ALLMI	2	46
Selenoprotein P	A0A151MWX0_ALLMI	1	45
Golgin subfamily A member 3 isoform A	A0A151NRP7_ALLMI	2	43
Serine incorporator 4	A0A151M668_ALLMI	1	42
Cleavage and polyadenylation specificity factor subunit 6 isoform B	A0A151PFC7_ALLMI	1	42
Protein AHNAK2	A0A151MYU6_ALLMI	1	41
Uncharacterized protein	A0A151M202_ALLMI	1	40
BTB/POZ domain-containing protein 7	A0A151P8X9_ALLMI	1	40
IgGFc-binding protein-like	A0A151MJC3_ALLMI	1	40
60S ribosomal protein L11 isoform A	A0A151MML3_ALLMI	1	40
Steroid 17-alpha-hydroxylase/17,20 lyase	A0A151NV24_ALLMI	2	39
Small G protein signaling modulator 1 isoform B	A0A151NSF2_ALLMI	1	39
Ig-like domain-containing protein	A0A151N4A7_ALLMI	1	39
TRAF family member-associated NF-kappa-B activator	A0A151M0A1_ALLMI	1	38
40S ribosomal protein S26	A0A151NSA5_ALLMI	1	38
Coagulation factor XII	A0A151NM16_ALLMI	1	38
Sushi domain-containing protein	A0A151NM16_ALLMI	1	37
Complement factor H-related protein 3-like	A0A151NBK6_ALLMI	1	36
Cadherin-1	A0A151P0T5_ALLMI	1	36
Histidine-rich glycoprotein	A0A151M7M7_ALLMI	1	34
TED_complement domain-containing protein	A0A151M7E8_ALLMI	1	34
Zinc finger castor-like protein 1 isoform C	A0A151N3T3_ALLMI	1	34
V-set domain-containing T-cell activation inhibitor 1-like	A0A151MVY5_ALLMI	1	34
C4b-binding protein alpha chain-like	A0A151MAQ5_ALLMI	1	33
Neuronal PAS domain-containing protein 3 isoform A	A0A151NKE6_ALLMI	1	33
Arf-GAP with coiled-coil, ANK repeat and PH domain-containing protein 1	A0A151N149_ALLMI	1	33
Tubulin alpha-2 chain	A0A151LY82_ALLMI	1	32

*Deiminated proteins from EVs were isolated by immunoprecipitation using the pan-deimination F95 antibody. The resulting F95-enriched eluate was then analyzed by LC-MS/MS and peak list files submitted to mascot. Alligator mississippiensis species-specific peptide sequence hits are listed (ALLMI), showing the number of sequences for protein hits and total score. Rows highlighted in pink indicate protein hits identified in plasma EVs only (for full details on protein hits, see Supplementary Table 2)*.

† *Ions score is −10 × Log(P), where P is the probability that the observed match is a random event. Individual ions scores >32 indicated identity or extensive homology (p < 0.05). Protein scores were derived from ions scores as a non-probabilistic basis for ranking protein hits*.

Deiminated proteins in alligator plasma were isolated by immunoprecipitation using the pan-deimination F95 antibody. The resulting F95-enriched eluate was then analyzed by LC-MS/MS and peak list files submitted to mascot. *Alligator mississippiensis* species-specific peptide sequence hits are listed (ALLMI), showing the number of sequences for protein hits and total score. Blue highlighted rows indicate protein hits identified in whole plasma only (for full details on protein hits, see Supplementary Table 1).

### Protein–Protein Interaction Network Identification of Deiminated Proteins in Plasma and EVs

**Figure 4:**
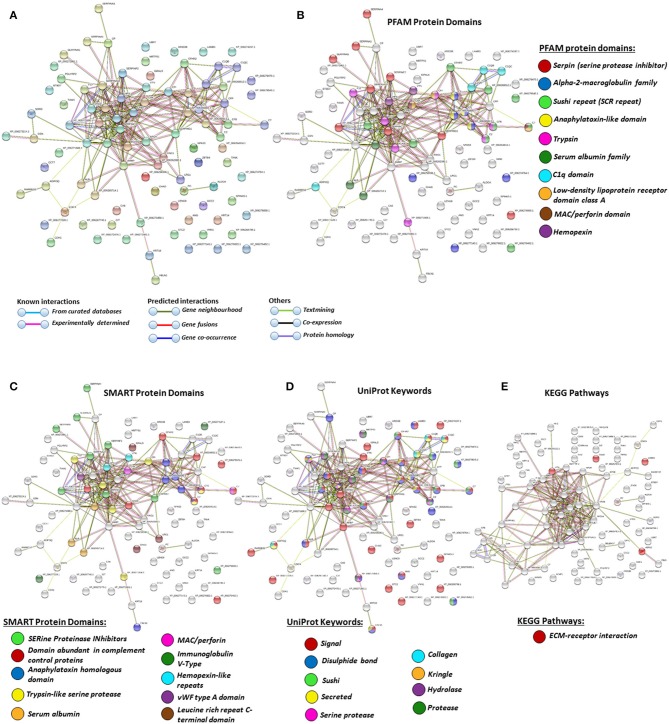
Protein–protein interaction networks of all deiminated proteins identified in alligator plasma. Reconstruction of protein–protein interactions based on known and predicted interactions in *Alligator mississippiensis*, using Search Tool for the Retrieval of Interacting Genes/Proteins (STRING) analysis. **(A)** Colored nodes represent query proteins and first shell of interactors. **(B)** Protein families (PFAM) protein domains relating to the identified proteins and reported in STRING are highlighted for: serpin, alpha-2-macroglobulin family, sushi repeat, anaphylatoxin-like domain, trypsin, serum albumin family, C1q domain, low-density lipoprotein (LDL)-receptor domain class A, membrane attack complex (MAC)/perforin domain, and hemopexin (see color code included in the figure). **(C)** Simple Modular Architecture Research Tool (SMART) protein domains relating to the identified proteins and reported in STRING are highlighted for serine proteinase inhibitors, domain abundant in complement control proteins, anaphylatoxin homologous domain, trypsin-like serine protease, serum albumin, MAC/perforin, Ig V-type, hemopexin-like repeats, von Willebrand factor (vWF) type A domain, and leucine-rich repeat C-terminal domain (see color code included in the figure). **(D)** UniProt keywords relating to the identified proteins and reported in STRING are highlighted for: signal, disulfide bond, sushi, secreted, serine protease, collagen, kringle, hydrolase, and protease (see color code included in the figure). **(E)** KEGG pathways relating to the identified proteins and reported in STRING are highlighted as follows: red = ECM-receptor interaction. Colored lines indicate whether protein interactions are identified via known interactions (curated databases, experimentally determined), predicted interactions (gene neighborhood, gene fusion, gene co-occurrence), or via text mining, coexpression, or protein homology (see the color key for connective lines included in the figure).

**Figure 5:**
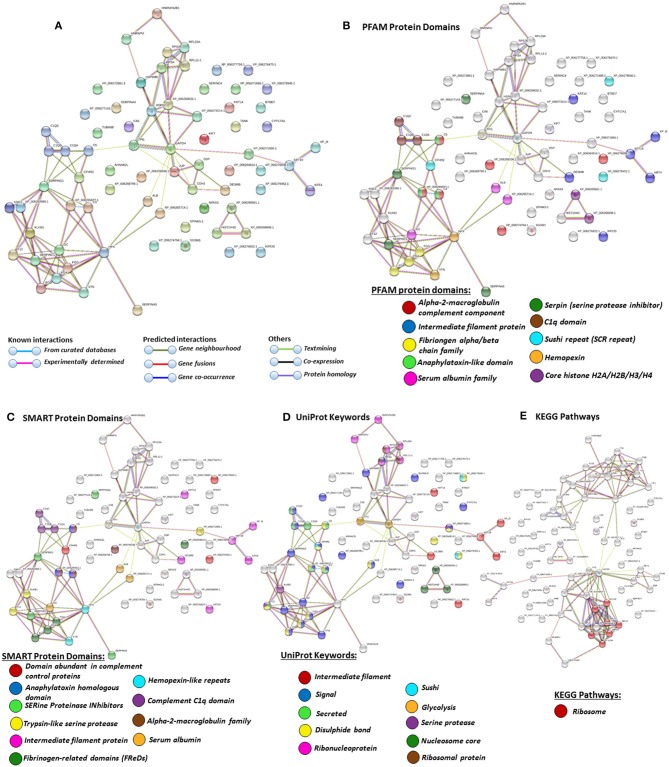
Protein–protein interaction networks of all deiminated proteins identified in plasma extracellular vesicles (EVs) of *Alligator mississippiensis*. Reconstruction of protein–protein interactions based on known and predicted interactions using the Search Tool for the Retrieval of Interacting Genes/Proteins (STRING) analysis. **(A)** Colored nodes represent query proteins and first shell of interactors. **(B)** Protein families (PFAM) protein domains relating to the identified proteins and reported in STRING are highlighted as follows: alpha-2-macroglobulin family, intermediate filament protein, fibrinogen chain family, anaphylatoxin-like domain, serum albumin family, serpin, C1q domain, sushi repeat, hemopexin, and core histones (see color code included in the figure). **(C)** SMART protein domains relating to the identified proteins and reported in STRING are highlighted as follows: domain abundant in complement control proteins, anaphylatoxin homologous domain, serine proteinase inhibitors, trypsin-like serine protease, intermediate filament protein, fibrinogen-related domains, hemopexin-like repeats, C1q domain, alpha-2-macroblobulin family, and serum albumin (see color code included in the figure). **(D)** UniProt keywords relating to the identified proteins and reported in STRING are highlighted as follows: intermediate filament, signal, secreted, disulfide bond, ribonucleoprotein, sushi, glycolysis, serine protease, nucleosome core, ribosomal protein (see color code included in the figure). **(E)** Kyoto Encyclopedia of Genes and Genomes (KEGG) pathways relating to the identified proteins and reported in STRING are highlighted as follows: red = ribosome. Colored lines indicate whether protein interactions are identified via known interactions (curated databases, experimentally determined), predicted interactions (gene neighborhood, gene fusion, gene co-occurrence), or via text mining, coexpression, or protein homology (see the color key for connective lines included in the figure).

**Figure 6:**
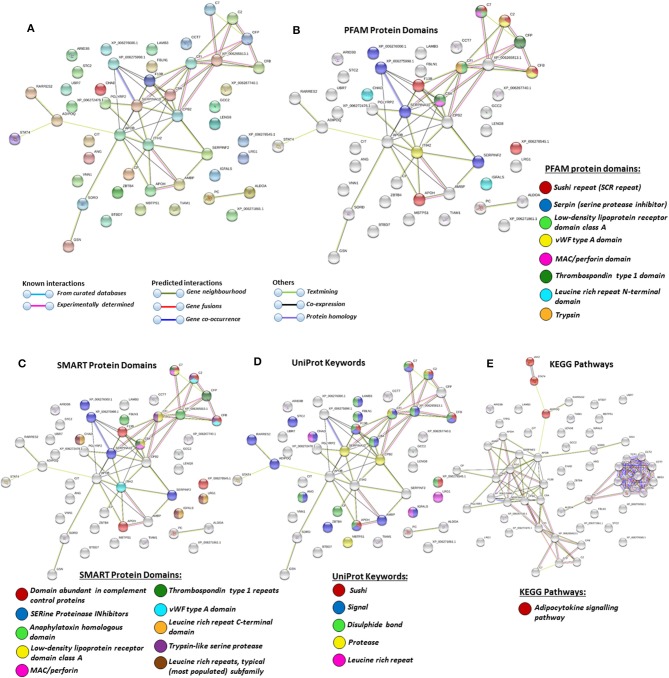
Protein–protein interaction networks of deiminated protein candidates identified in alligator plasma only (not identified in extracellular vesicles (EVs). Reconstruction of protein–protein interactions based on known and predicted interactions using the Search Tool for the Retrieval of Interacting Genes/Proteins (STRING) analysis. **(A)** Colored nodes represent query proteins and first shell of interactors. **(B)** Protein families (PFAM) protein domains relating to the identified proteins and reported in STRING are highlighted as follows: sushi repeat, serpin, low-density lipoprotein (LDL)-receptor domain class A, von Willebrand factor (vWF) type A domain, membrane attack complex (MAC)/perforin domain, thrombospondin type 1 domain, leucine-rich repeat N-terminal domain, and trypsin (see color code included in the figure). **(C)** Simple Modular Architecture Research Tool (SMART) protein domains relating to the identified proteins and reported in STRING are highlighted as follows: domain abundant in complement control proteins, serine proteinase inhibitors, anaphylatoxin homologous domain, LDL-receptor domain class A, MAC/perforin, thrombospondin type 1 repeats, vWF type A domain, leucine-rich repeat C-terminal domain, trypsin-like serine protease, leucine-rich repeats (see color code included in the figure). **(D)** UniProt keywords relating to the identified proteins and reported in STRING are highlighted as follows: sushi, signal, disulfide bond, protease, leucine-rich repeat (see color code included in the figure). **(E)** Kyoto Encyclopedia of Genes and Genomes (KEGG) pathways relating to the identified proteins and reported in STRING are highlighted as follows: red = adipocytokine signaling pathway.

**Figure 7:**
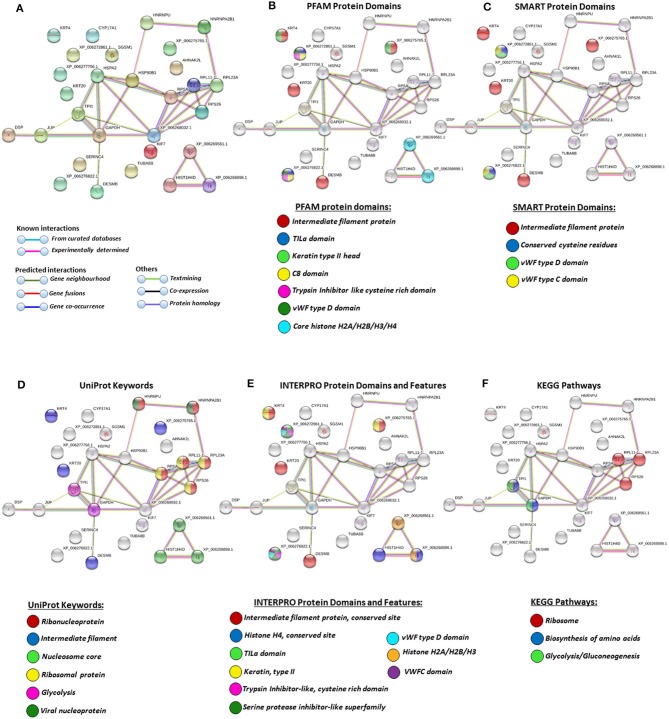
Protein–protein interaction networks of deiminated protein candidates identified in alligator extracellular vesicles (EVs) only (not identified in total plasma). Reconstruction of protein–protein interactions based on known and predicted interactions using the Search Tool for the Retrieval of Interacting Genes/Proteins (STRING) analysis. **(A)** Colored nodes represent query proteins and first shell of interactors. **(B)** Protein families (PFAM) protein domains relating to the identified proteins and reported in STRING are highlighted as follows: intermediate filament protein, TILa domain, keratin type II head, C8 domain, trypsin inhibitor-like cysteine-rich domain, von Willebrand factor (vWF) type D domain, core histones H2A/H2B/H3/H4 (see color code included in the figure). **(C)** Simple Modular Architecture Research Tool (SMART) protein domains relating to the identified proteins and reported in STRING are highlighted as follows: intermediate filament protein, conserved cysteine residues, vWF type D domain, and vWF type C domain (see color code included in the figure). **(D)** UniProt keywords relating to the identified proteins and reported in STRING are highlighted as follows: ribonucleoprotein, intermediate filament, nucleosome core, ribosomal protein, glycolysis, and viral nucleoprotein (see color code in legend). **(E)** INTERPRO protein domains and features: intermediate filament protein conserved site, histone H4, TILa domain, keratin type II, trypsin inhibitor-like cysteine-rich domain, serine protease inhibitor-like superfamily, vWF type D domain, histone H2A/H2B/H3, von Willebrand factor type C (VWFC) domain (see color code included in the figure). **(F)** Kyoto Encyclopedia of Genes and Genomes (KEGG) pathways relating to the identified deiminated proteins and reported in STRING are highlighted as follows: ribosome, biosynthesis of amino acids, and glycolysis/gluconeogenesis.

For the prediction of protein–protein interaction networks of these deimination candidate proteins, the protein ID lists for plasma and plasma EVs, respectively, were submitted to STRING analysis (https://string-db.org/) (Figures 4–7). Protein interaction networks were based on known and predicted interactions and represent all deiminated proteins identified in plasma (Figure 4), all deiminated proteins identified in EVs (Figure 5), as well as deiminated proteins identified in plasma only (Figure 6) or in EVs only (Figure 7). The protein–protein interaction (PPI) enrichment *p*-value for all deiminated proteins identified in alligator plasma (based on protein identifier sequences) was found to be *p* < 1.0 × 10^−16^, and for all deiminated proteins identified in the plasma-derived EVs, the PPI enrichment *p*-value was also found to be *p* < 1.0 × 10^−16^ (Figures 4, 5). For deiminated proteins identified in plasma only (but not EVs), the PPI enrichment *p*-value was also *p* < 1.0 × 10^−16^ (Figure 6), while for deiminated proteins identified specifically in EVs, the PPI enrichment *p*-value was *p* = 4.83 × 10^−8^ (Figure 7). This indicates that, in all cases, the identified protein networks have significantly more interactions than expected for a random set of proteins of similar size, drawn from the genome. The Kyoto Encyclopedia of Genes and Genomes (KEGG) pathways related to deiminated proteins identified as deiminated in whole plasma only, related to the adipocytokine signaling pathway (Figure 6E), based on STRING analysis for *A. mississippiensis*, while deiminated proteins identified as deiminated in EVs only belonged to KEGG pathways for ribosome, biosynthesis of amino acids, and glycolysis/gluconeogenesis (Figure 7E). Protein families (PFAM) protein domains differed for deamination-specific proteins in plasma compared to in EVs (Figures 6B, 7B), as did Simple Modular Architecture Research Tool (SMART) protein domains, which for plasma showed serpin, trypsin, and complement-related domains [von Willebrand factor (vWF) and membrane attack complex (MAC)/perforin domains], while EVs showed also core histone domains (Figures 6C, 7C). STRING analysis for UniProt keywords, INTERPRO [http://www.ebi.ac.uk/interpro/; (92)] protein domains and features are furthermore highlighted for plasma and plasma EVs (Figures 4–7).

### Phylogeny Tree for American Alligator PADs Compared to Human PADs

**Figure 8:**
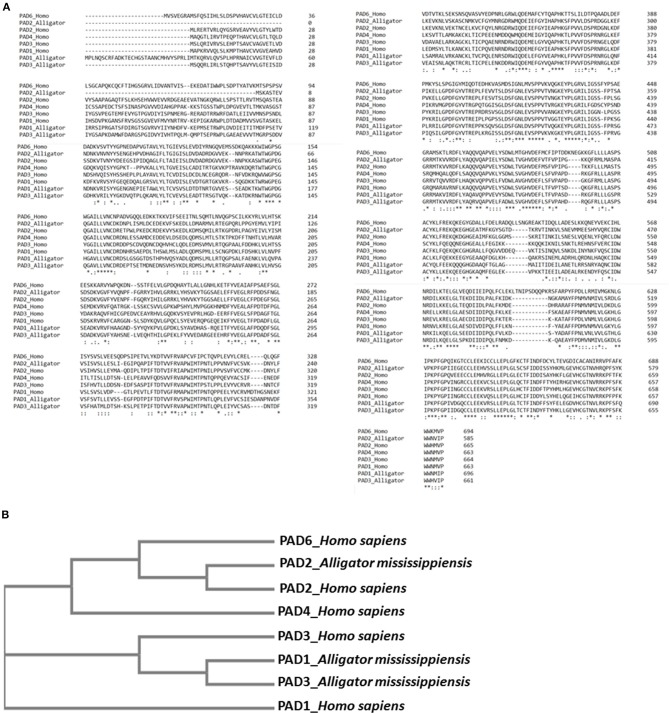
Sequence alignment and phylogeny tree for alligator peptidylarginine deiminases (PADs) compared with human PADs. **(A)** Multiple sequence alignment of reported (predicted) alligator PAD sequences PAD1 (XP_006259278.3), PAD2 (XP_019355592.1), and PAD3 (XP_014457295.1) isozymes, compared with human PAD isozyme protein sequences PAD1 (NP_037490.2), PAD2 (NP_031391.2), PAD3 (NP_057317.2), PAD4 (NP_036519.2), and PAD6 (NP_997304.3), using Clustal Omega. **(B)** A neighbor joining tree is shown for phylogenetic clustering of the reported (predicted) alligator PAD1 (XP_006259278.3), PAD2 (XP_019355592.1), and PAD3 (XP_014457295.1) isozymes, compared with human PAD isozyme protein sequences PAD1 (NP_037490.2), PAD2 (NP_031391.2), PAD3 (NP_057317.2), PAD4 (NP_036519.2), and PAD6 (NP_997304.3), using Clustal Omega.

A phylogeny tree for American alligator reported and predicted PAD sequences (PAD1, 2, and 3) compared to human PADs 1, 2, 3, 4, and 6, using Clustal Omega, revealed the closest relationship between alligator PAD2 with human PAD2 (Figure 8). This correlates with the strongest cross-reaction detected with the antihuman PAD2 antibody in both alligator plasma and plasma EVs (Figure 2).

## Discussion

The current study is the first to profile deiminated proteins in plasma and EVs of American alligator (*A. mississippiensis*), and the first such study of a non-avian diapsid (extant reptiles). F95 enrichment revealed a range of immunological, metabolic, and gene regulatory proteins as candidates for this posttranslational modification, therefore indicating hitherto underrecognized modes for protein moonlighting of these proteins in alligator physiology and immunity. PAD proteins were identified in alligator plasma via cross-reaction to antibodies raised against human PAD isozymes (PAD2, 3, and 4), which were previously shown to cross-react with PADs with diverse taxa, and such detection is in accordance with that PADs have previously reported in the alligator genome (PADI1, Gene ID: 102574884; PADI2, Gene ID: 102575591; PADI3, Gene ID: 102574651). The antihuman PAD2 antibody showed strongest cross-reaction with alligator plasma proteins at a predicted size of 70–75 kDa for PAD proteins, also in the plasma EVs, and this is in accordance with previous findings reporting PAD2 to be the most phylogenetically conserved isozyme (14, 56, 58, 76, 77, 79), as also confirmed by the cladogram constructed based on sequence alignment of predicted and reported protein sequences for alligator and human PAD isozymes (Figures 8A,B). Furthermore, the difference detected in cross-reaction with antihuman PAD2, PAD3, and PAD4 antibodies (note that neither antihuman PAD1 nor PAD6 antibodies were assessed here) in whole plasma compared with plasma EVs, as PAD4 did not show positive in the EVs, may be associated with the differences observed in deiminated protein targets in plasma vs. plasma EVs. Preferences for the different PAD isozymes against cellular substrates is indeed known (93). The presence of deiminated histone H3 (citH3), which sometimes is used as a marker of NETosis was also identified here by Western blotting in alligator plasma, providing the first evidence of PAD-mediated NETosis mechanisms in reptiles, although other circulating histones have previously been identified in crocodilian blood (94). While NETosis has been shown to be a phylogenetically conserved mechanism from fish to mammals (95), the only other studies on NETosis related to reptiles have been investigations on snake venom, showing that Indian saw-scaled viper (*Echis carinatus*) venom induces neutrophil extracellular trap (NET) formation in host tissue, through which it contributes to tissue destruction of the affected area (96, 97). It has to be noted though that further evaluation of NETosis in alligator plasma will need to be performed, as the direct link between histone citrullination/deimination and NETosis has been challenged (98).

A number of alligator species-specific deiminated protein candidates were identified in both plasma and plasma-derived EVs, using F95 enrichment in tandem with LC-MS/MS analysis. This analysis revealed some key metabolic and immune-related proteins, with 53 characterized common deiminated proteins in plasma and EVs, while 59 characterized deiminated protein hits were specific for plasma and 24 characterized deiminated protein hits were specific for EVs. Upon assessment of protein–protein interaction networks using STRING analysis, the PPI enrichment *p*-value for all deiminated proteins identified in alligator plasma and in plasma EVs, as well as for deiminated proteins identified either in plasma or EVs only, indicated that the identified protein networks have significantly more interactions than expected for a random set of proteins of similar size, drawn from the genome, and that the proteins are at least partially biologically connected, as a group (Figures 4–7).

In plasma, deiminated protein targets identified belonged to KEGG pathways for extracellular matrix (ECM)-receptor interaction and adipocytokine signaling pathway (Figures 4E, 6E). ECM-receptor interactions control both directly and indirectly a range of cellular activities including migration, adhesion, differentiation, apoptosis, and proliferation and have been studied in cancer, also at the transcriptome level (99). KEGG pathways for ECM-receptor interaction have, for example, been previously identified to be enriched in EVs of mesenchymal stem cells (100), but regulation via posttranslational deimination has not been investigated. A recent study identified enrichment of deiminated proteins in KEGG pathways for ECM-receptor interactions in the fin whale, also long-lived cancer-resistant animal (75). Interestingly, deimination of KEGG pathways of ECM-receptor interactions has also been identified by our group in the wandering albatross (*Diomedea exulans*) (80), which emphasizes the phylogenetic relationship between reptiles and birds, while exact phylogenetic reconstructions can though vary according to genomic or protein parameters used, partly also due to convergent evolution (101).

The adipocytokine signaling pathway plays important roles in metabolic regulation and is involved in a range of pathologies including insulin resistance and type II diabetes (102, 103). Adiponectin is one of the cytokines secreted by adipocytes and was here identified in alligator whole plasma only. Adiponectin has key functions in regulating glucose (104–106) and is also linked to regenerative functions (107), longevity (108), cancer (109), and myopathies (110). It has recently been identified as a deimination candidate in several taxa with unusual metabolism including the llama (*Lama glama*), the naked mole rat (*Heterocephelus glabe*r), and orca (*Orcinus orca*) (75, 76, 79).

In alligator plasma EVs, F95 target proteins identified as deiminated proteins belonged to ribosomal, biosynthesis of amino acids, and glycolysis/glyconeogenesis KEGG pathways (Figures 5E, 7E). Furthermore, STRING analysis was carried out on deiminated protein hits found specifically in EVs only as well as for those only found in plasma, excluding overlapping protein hits (Figures 6, 7). This revealed enrichment of deiminated proteins in plasma linked to KEGG pathways of adipocytokine signaling (Figure 6), while in EVs, pathways related to ribosomal, biosynthesis of amino acids, and glycolysis/gluconeogenesis were enriched in deiminated proteins (Figure 7). In EVs, histone pathways were also enriched, alongside vWF and intermediate filament protein domains, and PFAM domains relating to the complement pathway, fibrinogen, serpins, anaphylatoxins, hemopexin, and serum albumin (Figures 5B–E, 7B–E).

KEGG ribosomal pathways are linked to cancer-associated processes (111), and putative regulation of these networks via deimination may therefore be of importance. Furthermore, in relation to KEGG pathways for biosynthesis of amino acids, deimination-mediated changes may be of considerable interest for comparative metabolic studies, particularly as amino acid assessment for mammalian metabolism and for research into aging and disease has received some attention (112). Glycolysis is of high relevance in metabolism and cancer, and pathways for glycolysis/gluconeogenesis, identified here in alligator plasma EVs, have previously been identified in cancer cells (27) and in several species of whale (75), which are long lived and cancer-resistant sea mammals. Interestingly, in the naked mole rat, also an animal with low metabolic rate, cancer resistance, and unusual longevity, deiminated proteins relevant to glycolysis were also found to be enriched in plasma EVs specifically (79). Whether deimination in these pathways identified here in alligator is of some relevance for the low metabolic rate and cancer resistance found in alligator (10) will remain to be further investigated.

Deiminated protein candidates involved in immune pathways for antipathogenic defenses, including complement related proteins, were found both in whole plasma and enriched in the plasma EVs, indicative of EV-mediated transport of deiminated protein components. This coincides with previous findings of unusual antimicrobial defenses of alligator, much higher than in human sera and likely to be complement dependent (113). Furthermore, alligator sera has also been found active against multidrug resistant bacteria such as *Acinetobacter baumanii* and *Klebsiella pneumoniae* (8), as well as amoeba (114).

A range of proteins from the complement cascade was indeed identified here as deiminated in alligator, and this has also recently been found in other species by our group (58, 73–79), including in avian species (80). The complement system bridges innate and adaptive immunity, participates in the clearance of necrotic and apoptotic cells, and forms part of the first lines of immune defenses against invading pathogens (115–119). Interestingly, properdin was here identified as deiminated in alligator plasma for the first time in any species, as this has not been shown to be a deimination candidate in other taxa studied so far. Properdin is a positive regulator of the alternative complement pathway (AP) and linked to multifaceted roles in inflammation and disease (120, 121). It is a plasma glycoprotein that stabilizes C3 and C5 convertases and initiates and positively regulates AP activity (120, 122, 123). Properdin-mediated complement activity contributes to innate and adaptive immune responses and tissue damage, and properdin has therefore also been a target for modulation in disease pathologies (120, 124). While properdin is a known glycoprotein, deimination of properdin is here described for the first time in any species and may shed a novel light on how properdin can take on its multifaceted roles, possibly also via such posttranslational changes.

The properdin target C3 was the most identified deiminated protein in the alligator plasma and EVs. C3 has recently been identified to be deiminated in our studies in a range of taxa (76, 77, 80). Furthermore, an abundance of deiminated complement components identified to be deiminated both in plasma and plasma EVs included, besides C3, complement receptor type-1, complement factor H, complement factor I, complement C1q subcomponent subunits A and C, C4b-binding protein-like, while C2, C7, factor B, and properdin were deiminated in whole plasma only. Deimination of the various complement components, except properdin, has recently been identified by our group in teleost and cartilaginous fish (58, 76, 78), camelids (77), cetaceans (75), and birds (80). These findings indicate hitherto understudied roles for posttranslational deimination in the known diversity of complement function throughout phylogeny (125–129). Indeed, as some of the antibacterial effects of crocodile serum have been linked to the complement system (5, 113), our findings suggest that protein deimination may play hitherto unidentified roles in the known unusual antimicrobial and anti-inflammatory function of alligator (5, 130, 131), including via EV transport in cellular communication, also playing roles in complement function in homeostatic processes. Ficolin-3 was furthermore identified to be deiminated in whole alligator plasma only and is a sugar pattern recognition molecule, which forms part of mammalian immune systems (132). Ficolin-3 can activate the complement system via the lectin pathway (133), plays roles in bacterial defenses (134, 135) and autoimmunity (136, 137) and is modulated in viral infections including HIV (138). Ficolin-3 has been associated with metabolic diseases including gestational, prediabetes, and type 2 diabetes (139, 140), and identified as biomarkers in axial spondyloarthritis (141) and as a prognostic biomarker for esophageal cancer (142). Studies on ficolins in reptiles are limited, besides putative roles for veficolins in reptile venom systems (143, 144) and for ficolin superfamily proteins as snake venom metalloproteinase inhibitors (145). Deimination of ficolin-3 has been previously identified only in the naked mole rat (79), a cancer- and hypoxia-resistant animal with unusual immunity and longevity. The roles of posttranslational deimination in regulation of ficolin-mediated mechanisms may therefore be of considerable interest, including in relation to inflammatory and oncogenic pathways.

Alpha-2-macroglobulin (alpha-2-M) was found to be deiminated in alligator plasma and plasma EVs and was identified as a PFAM protein domain in both. It clears active proteases from tissue fluids and forms part of innate immunity (146). A range of protease inhibitors and proteases were furthermore identified in both plasma and plasma EVs including plasma protease C1 inhibitor (in plasma and plasma EVs) and protein Z-dependent protease inhibitor (in plasma) and membrane-bound transcription factor site-1 protease (in plasma). Alpha-2-M is conserved throughout phylogeny from arthropods to mammals and closely related complement proteins C3, C4, and C5, which are also thioester-containing proteins (115, 147, 148). Crocodilian alpha-2-M is homologous to human alpha 2-M or chicken ovomacroglobulin and has been assessed in Cuban crocodile (*Crocodylus rhombifer*) (149), while its structure has furthermore been assessed by electron microscopy in *Crocodylus siamensis* (150). While structural changes of alpha-2- and ovomacroglobulin have been assessed to some extent (151), including the identification of three intramolecular thiol ester bonds in crocodilian ovomacroglobulin, which display differential stability against external perturbations (152), structural or functional changes mediated via posttranslational deimination have not been assessed. The deimination of alpha-2-M has recently been identified by our group in camelid and birds (77, 80).

Serpin (serine proteinase inhibitor) PFAM and SMART domains were identified as deiminated both in alligator plasma and EVs, with specific targets identified being serpin peptidase inhibitor. Such deimination may provide a novel insight into utilizing serpin-based peptides as antimicrobials against multidrug resistant pathogens (6, 7). Deimination of serpin may also be important in the human rheumatoid arthritis citrullinome, where deimination has previously been shown to modulate protease activity, resulting in downstream effects on serpin-regulated pathways (29). Furthermore, a range of apolipoproteins was identified to be deiminated in alligator whole plasma. Apolipoproteins have antimicrobial activity against a range of pathogenic bacteria (153–156), including in alligator, and have been tested for use against several multidrug-resistant bacteria (6, 7). Various apolipoproteins have recently been identified as deimination protein candidates by our group in a range of taxa (56, 58, 77), including in pelagic seabirds (80).

Hemoglobin, which was identified here as being deiminated in alligator plasma, has, alongside crude leukocyte extract and plasma, been found to have antioxidant and anti-inflammatory activities in Chinese crocodile (*Crocodylus siamensis*) (131). Furthermore, viral nucleoprotein was identified as a UniProt keyword connected to deiminated proteins specific to EVs only, which may be of relevance in the light of antiviral activity of alligator serum against enveloped viruses, including human immunodeficiency virus type 1 (HIV), West Nile virus (WNV), and herpes simplex virus type 1 (HSV-1) (9). Interestingly, serine incorporator 4 was identified as deiminated in alligator plasma EVs only, and serine incorporator proteins have recently been identified as novel host restriction factors implicated in HIV-1 replication (157). This highlights a hitherto unrecognized posttranslational control mechanism of various proteins involved in antiviral responses.

The presence of deiminated histone H2A and H4 was identified in alligator EVs only. This may be of some interest, as extracellular histones H2A and H4 in crocodile blood have indeed been identified to act as inhibitors of viral (HIV) infection *in vitro* (94). While some studies in reptiles have assessed histone deacetylation, methylation, and histone variants (158–160), as well as linking histone methylation to anoxia survival in turtles (161), studies on posttranslational deimination of histones is lacking in non-avian reptiles. Histone H3 deimination has been previously identified inked to inflammatory responses during CNS regeneration in the chicken (*Gallus gallus*) (40) and in hypoxic responses during CNS repair (41). Histone deimination is also known to be involved in epigenetic regulation involved in cancer (17, 20). Interestingly, a similar EV export of deiminated histones as observed in alligator plasma here was recently identified in the naked mole rat, also an unusually long-lived and cancer-resistant animal (79). Indeed, the use of non-mammalian model organisms in epigenetic research has been highlighted, including in reptiles (162), and roles for histone modifications, including deimination identified here, may be of interest, as crocodilians are also long lived, cancer and hypoxia resistant, and furthermore show unusually resistant antipathogenic responses (4, 163).

AHNAK2 is a nucleoprotein and was identified to be deiminated in alligator plasma EVs only. AHNAK is a multifaceted proteins with roles in cell architecture and migration, blood–brain barrier formation, regulation of cardiac calcium channels, and repair of muscle membranes (164). Furthermore, roles in cancer are implicated, and AHNAK has been shown to facilitate EV release in mammary carcinoma cells (165), therefore playing critical roles in EV communication in the tumor environment. AHNAK has been identified as a biomarker in several, including metastatic, cancers (166–169) and linked to drug resistance in cancer in association with viral infection (170). AHNAK has been is also related to stress-induced secretion of FGF1—a growth factor regulating carcinogenesis, angiogenesis, and inflammation (171) and to inherited peripheral neuropathy (172). AHNAK was previously identified to be deiminated in aggressive glioblastoma cells by our group (27, 61). The deimination of AHNAK identified here specifically in alligator plasma EVs may play some roles in antipathogenic resistance but will remain to be further investigated, also in relation to human pathologies.

Protein adrenomedullin binding protein-1 (AMBP) was identified as deiminated in whole alligator plasma only. AMBP is a plasma protein that binds adrenomedullin and acts as an important modulator in the biphasic septic response, including during the progression of polymicrobial sepsis (173, 174). Insights into posttranslational regulation of AMBP via deimination may therefore be of importance for the management of sepsis and is of great interest in the light of the unusual antimicrobial properties of alligator plasma.

Various Ig proteins and Ig superfamily members were identified here to be deiminated in alligator plasma and plasma EVs, confirming that Igs can be exported via EVs. Ig proteins identified common in whole plasma and plasma EVs were IgGFc-binding protein-like, Ig lambda light chain variable region, Ig epsilon chain constant region, and Ig-like domain-containing protein, while Ig heavy chain variable region was identified as deiminated only in whole plasma. Several studies have assessed Igs in crocodilians including IgH subclass-encoding genes and IgM subclass switching (175), IgA evolution (176), and analysis of Ig light (L) chains, revealing a highly diverse IgL gene repertoire (3). Posttranslational modifications and such contribution to Ig diversity remains though to be studied. We have previously confirmed posttranslational deimination if Igs in several taxa, including shark, camelid, and birds (76, 77, 80), and furthermore reported EV-mediated transport of Igs in shark and camelid (76, 77). Igs play key roles in adaptive immunity and have been extensively studied in diverse taxa. Posttranslational deimination of Igs and downstream roles in Ig function have though received little attention, until recent studies in teleosts and cartilaginous fish (56, 58), camelids (77) and cetaceans (75). In human patients with rheumatoid arthritis (RA) and bronchiectasis, it has been reported that the IgG Fc region is posttranslationally deiminated (177). In the light of growing interest in elucidating Ig diversity throughout the phylogenetic tree (178–183), our finding of deimination of crocodilian Igs in the current study highlights a novel concept of diversification of Igs via such posttranslational deimination.

T-lymphoma invasion and metastasis-inducing protein 1 (TIAM1) was here identified as deiminated in alligator plasma. It is important in the regulation of cell membrane dynamics (184), involved in the regulation of phagocytosis (185) and bacterial cell invasion in the host (186). TIAM1 has also been shown to play roles in neuronal responses to oxygen and glucose deprivation (187) and has been linked to mitochondrial dysfunction in diabetic retinopathy (188, 189) as well as to retinoblastoma (190). TIAM1 promotes chemoresistance and tumor invasiveness (191), and its expression levels are positively correlated to with poor prognosis in solid cancers (192). It is associated with histone methyltransferases in epigenetic regulation for cancer progression (193). TIAM1-mediated networks are also implicated in neuroblastoma, and therefore, strategies to regulate TIAM1 have been highlighted (194). TIAM1-regulated pathways have furthermore been highlighted as targets in autoimmunity (195), including in islet β cells in health and diabetes (196, 197). While phosphorylation of TIAM1 has been studied in relation to neurological disease (198), the posttranslational deimination of TIAM1 identified here in alligator has not been identified in any species so far to our knowledge. Such deimination-mediated changes may indicate regulatory pathways of this protein with respect to hypoxia tolerance, cancer, and autoimmune pathologies as well as host–pathogen interactions. Furthermore, posttranslational deimination of TIAM may be of some relevance in the context of utilizing TIAM pathways for the generation of optogenetic tools (184).

Exostosin-like-1 (EXTL-1) was here deiminated in whole alligator plasma only. It belongs to a family of glycosyltransferases, involved in heparin sulfate and heparin biosynthesis as well as acting as tumor suppressors (199, 200). EXTL-1 has furthermore been found to have important functions in regulation of tau uptake in relation to neurodegeneration (201). The deimination of EXTL-1 identified here in whole alligator plasma may therefore be of some relevance regarding regulation of its function via such posttranslational change and has not been described in any species so far to our knowledge.

Selenoprotein P (Sepp1) was identified to be deiminated in whole alligator plasma only. It is a plasma glycoprotein, secreted mainly from liver, as well as other tissues, and it contains most of mammalian plasma selenium (202, 203). It has antioxidant properties (202). Sepp1 has roles in homeostasis and in the distribution of selenium (203). Sepp1 is believed to have phylogenetically appeared in early metazoan species, as terrestrial animals have fewer selenoproteins than marine animals, and this may partly be reflected in different functions (204). Selenoproteins have been studied in a range of non-mammalian vertebrates including agnathans and birds (205), but no studies in particular have been carried out in alligator. While Sepp1 is known to be glycosylated, its deimination has not been studied besides being recently identified in whales (75).

l-Lactate dehydrogenase was found deiminated in EVs only, and lactate dehydrogenase has previously been identified to be a hepatic biomarker in American alligator (206). l-Lactate controls apoptosis and autophagy in tumor cells (207) and plays important roles in the tumor microenvironment, including under hypoxic conditions, and lactate dehydrogenase metabolism has been identified as a target to overcome resistance to immune therapy of tumors (208). l-Lactate dehydrogenase has previously been identified by our group to be deiminated in glioblastoma cells (27) and in plasma of naked mole rat (79) and in minke whale (75), both long-lived and cancer-resistant animals. Whether posttranslational deimination may play roles in the regulation of lactate dehydrogenase metabolism, and therefore affect pro- or anticancerous responses, remains to be investigated.

Stanniocalcin-2 (STC2) was identified as deiminated in alligator whole plasma and has not been identified as a deimination candidate in other taxa studied so far. Stanniocalcin is a secreted glycoprotein, originally studied in fish as hormone regulator of serum calcium levels (209). It is expressed in a wide range of tissues and regulates various biological processes including lipid and glucose metabolism (210, 211), the growth hormone-insulin-like growth factor axis (212), as well as cellular calcium and phosphate homeostasis (213, 214). STC2 is related to growth restriction (209) and organomegaly when overexpressed at the genetic level (215). It is upregulated in response to metabolic stresses, including hypoxia conditions (216), and forms part of the unfolded protein response (217). STC2 is also a tumor marker for several cancers as well as possibly involved in metastasis (218, 219). Studies on STC2 have not been performed in reptiles, while expression patterns have been assessed in avian muscle and joint development (220). Roles for posttranslational deimination of STC2 remain to be understood both in reptile physiology as well as in relation to human pathologies.

Glyceraldehyde-3-phosphate dehydrogenase (GAPDH) was here identified as deiminated in alligator plasma EVs only. GAPDH is important in glycolysis, where it has key metabolic functions, while it also has pleiotropic non-metabolic functions including in mitochondrial regulation in apoptosis, in axonal transport, and in transcription activation (221–224). Furthermore, a range of moonlighting functions has been identified for GAPDH, including roles in iron metabolism (225), while it is also associated to various pathologies (223). In crocodilians, GAPDH has been studied in the muscle of caiman (226). GAPDH has been shown to be regulated via some posttranslational modifications (224, 227, 228) and was recently identified as a deimination candidate by our group in cancer (61), as well as in whales (75) and to form part of deiminated protein EV cargo in naked mole rat plasma (79). Deimination of GAPDH may contribute to its multifaceted physiological functions, and the identification of its deimination in several taxa with unusual metabolism and cancer resistance is of considerable interest.

Desmoplakin was found deiminated in alligator plasma EVs only in the current study. It is an important component of desmosomal cell–cell junctions and also involved in the coordination of cell migration as well as in maintaining integrity of the cytoskeletal intermediate filament network (229). In the *Xenopus laevis* embryo, it is required for morphogenesis and for epidermal integrity (230). A range of allergies have been linked to mutations in desmoplakin, as well as metabolic wasting (SAM) syndrome and severe dermatitis (231). Desmoplakin has also roles in Carvajal syndrome, relating to hair abnormalities and altered skin (232). It is furthermore related to heart diseases, including cardiomyopathies (233), and found to interact with desmin, which is related to cardiomyopathies (234, 235). Other roles for desmosomal proteins relate to both tumor-suppressive and tumor-promoting functions, which depends on the type of cancer, and they can furthermore regulate cell migration, differentiation, proliferation, and apoptosis, as well as impacting sensitivity to treatment in different types of cancers (236). Interestingly, desmoplakin has recently been identified in deiminated form in camelid EVs (77), therefore indicating that enrichment of deiminated desmoplakin in EVs is found across taxa. As the functions of desmosomal proteins are not fully understood in cancer or metastasis, the current identification of deimination in alligator EVs here may be of considerable interest, also due to important roles of EV-mediated communication in the preparation of the metastatic niche. This may further current understanding of the diverse functional ability of desmoplakin, via such posttranslational modification.

Heat-shock protein mitochondrial was found to be deiminated in alligator plasma EVs only. Heat-shock proteins are phylogenetically conserved chaperone proteins involved in protein folding, protein degradation, and the stabilization of proteins against heat stress (237, 238). Heat-shock proteins are involved in mitochondrial metabolic reprogramming and therefore of importance in pro- and antioncogenic pathways (239). Heat-shock proteins are also involved in inflammation, can act as damage-associated molecular patterns (DAMPs) (240), and have furthermore been identified to be deiminated in human autoimmune disease (241). Previously, some heat-shock proteins have been verified as deimination candidates by our group in teleost fish, camelid, and cetaceans (56, 75, 77), as well as in plasma EVs of naked mole rat (79). Finding posttranslational deimination of heat-shock proteins throughout phylogeny supports translational investigations between species to further current understanding of their diverse physiological and pathobiological functions.

In the current study, we report for the first time deimination signatures of plasma and plasma-derived EVs of American alligator. Posttranslational deimination of major key immune and metabolic factors was identified and related to pathways ECM-receptor interaction, ribosome, adipocytokine signaling, biosynthesis of amino acids, and glycolysis/gluconeogenesis. The reported findings highlights posttranslational deimination as an important factor in protein moonlighting, including via EV-mediated transport. Our findings furthermore contribute to a growing body of research investigating posttranslational regulation of antipathogenic and anticancerous, as well as metabolic and inflammatory pathways via posttranslational deimination and EV-mediated transport of such modified proteins. EV research in comparative animal models is an understudied but recently growing field, and this is, to our knowledge, the first characterization of EVs and associated deiminated protein cargo in a reptile. As PADs have been identified as a major player in the regulation of EV release (59–61) including in host–pathogen interactions (62, 82), such PAD-mediated contributions to cell communication remain to be further investigated both in response to physiological and pathophysiological changes, as well as in zoonotic diseases.

## Conclusion

This is the first study to assess protein deimination profiles in plasma and EVs of a non-avian reptile, using *A. mississippiensis* as a model organism. KEGG pathways identified to be specific to deiminated proteins in whole plasma related to adipocytokine signaling, while KEGG pathways of deiminated proteins specific to EVs included ribosome, biosynthesis of amino acids, and glycolysis/gluconeogenesis pathways, as well as core histones. This highlights roles for EV-mediated export of deiminated protein cargo functioning in metabolism and gene regulation, also related to cancer. The identification of posttranslational deimination and EV-mediated communication in alligator plasma revealed here contributes to current understanding of protein moonlighting functions and EV-mediated communication in these ancient reptiles, providing novel insight into their unusual immune systems and physiological traits. Comparative studies in long-lived animals with unusual immune and metabolic functions, including cancer, antiviral and antibacterial resistance, may be of translational value for furthering current understanding of mechanisms underlying such pathogenic pathways, including via the diversification of protein function by posttranslational deimination.

## Data Availability Statement

All datasets generated for this study are included in the article/Supplementary Material.

## Ethics Statement

The animal study was reviewed and approved by The Texas A&M Institutional Care and Use Committee. Sample collection was conducted under Texas A&M Institutional Animal Care and Use Protocol #2015–0347.

## Author Contributions

MC: resources, validation, and writing—review and editing. IK: methodology, resources, visualization. LP: resources, methodology. SL: conceptualization, data curation, formal analysis, funding acquisition, investigation, methodology, project administration, resources, validation, visualization, Writing—original draft, and writing–review and editing.

## Conflict of Interest

The authors declare that the research was conducted in the absence of any commercial or financial relationships that could be construed as a potential conflict of interest.

## Supplementary Material

Supplementary Table 1 F95-enriched proteins from plasma of American alligator (*Alligator mississippiensis*). Full details of protein hits are reported.

Supplementary Table 2 F95-enriched proteins from plasma-EVs of American alligator (*Alligator mississippiensis*) plasma. Full details of protein hits are reported.
